# Molecular Mechanisms of the Deregulation of Muscle Contraction Induced by the R90P Mutation in Tpm3.12 and the Weakening of This Effect by BDM and W7

**DOI:** 10.3390/ijms22126318

**Published:** 2021-06-12

**Authors:** Yurii S. Borovikov, Daria D. Andreeva, Stanislava V. Avrova, Vladimir V. Sirenko, Armen O. Simonyan, Charles S. Redwood, Olga E. Karpicheva

**Affiliations:** 1 Laboratory of Molecular Basis of Cell Motility, Institute of Cytology of the Russian Academy of Sciences, 4 Tikhoretsky Ave., 194064 Saint Petersburg, Russia; daria1.andreeva@gmail.com (D.D.A.); avrova@rambler.ru (S.V.A.); sirw@mail.ru (V.V.S.); simonyan_armen@mail.ru (A.O.S.); olexiya6@ya.ru (O.E.K.); 2 Radcliffe Department of Medicine, University of Oxford, John Radcliffe Hospital, Oxford OX3 9DU, UK; credwood@well.ox.ac.uk

**Keywords:** tropomyosin, mutations in tropomyosin, muscle weakness, congenital myopathy, Ca^2+^-sensitivity of myofilament, ATPase activity of myosin, 2,3-butanedione monoxime (BDM), n-(6-aminohexyl) 5-chloro-1-naphthalenesulfonamide (W7)

## Abstract

Point mutations in the genes encoding the skeletal muscle isoforms of tropomyosin can cause a range of muscle diseases. The amino acid substitution of Arg for Pro residue in the 90th position (R90P) in γ-tropomyosin (Tpm3.12) is associated with congenital fiber type disproportion and muscle weakness. The molecular mechanisms underlying muscle dysfunction in this disease remain unclear. Here, we observed that this mutation causes an abnormally high Ca^2+^-sensitivity of myofilaments in vitro and in muscle fibers. To determine the critical conformational changes that myosin, actin, and tropomyosin undergo during the ATPase cycle and the alterations in these changes caused by R90P replacement in Tpm3.12, we used polarized fluorimetry. It was shown that the R90P mutation inhibits the ability of tropomyosin to shift towards the outer domains of actin, which is accompanied by the almost complete depression of troponin’s ability to switch actin monomers off and to reduce the amount of the myosin heads weakly bound to F-actin at a low Ca^2+^. These changes in the behavior of tropomyosin and the troponin–tropomyosin complex, as well as in the balance of strongly and weakly bound myosin heads in the ATPase cycle may underlie the occurrence of both abnormally high Ca^2+^-sensitivity and muscle weakness. BDM, an inhibitor of myosin ATPase activity, and W7, a troponin C antagonist, restore the ability of tropomyosin for Ca^2+^-dependent movement and the ability of the troponin–tropomyosin complex to switch actin monomers off, demonstrating a weakening of the damaging effect of the R90P mutation on muscle contractility.

## 1. Introduction

Muscle contraction is generated by the interaction of myosin cross-bridges with actin filaments and ATP. The myosin cross-bridges periodically bind to the actin filament [1] and, during force generation, pass through several conformational states, the so-called “strong” and “weak” forms of myosin binding to actin [2]. In striated muscle, the interaction of myosin with actin is regulated by the movements of the tropomyosin–troponin (Tpm–TN) complex, located on the actin filaments, in response to a change in the intracellular Ca^2+^ concentration. Both the structural and biochemical data suggest that tropomyosin strands can occupy three different positions on actin (so-called “blocked” or calcium-free, “closed” or calcium-induced, and “open” or myosin-induced), depending on the presence or absence of TN, myosin, and Ca^2+^ [2,3,4,5,6,7,8,9]. It is suggested that the electrostatic nature of the actin–tropomyosin interaction and the flexibility of actin and Tpm [3,6] can explain the dynamic displacement of Tpm relative to the outer and inner domains of actin (between the blocked, closed, and open positions) during contraction [4,5,6,7,8,9]. The change in the position of the Tpm strands relative to the inner domains of actin is due to the difference between tropomyosin and F-actin in their bending flexibility (therefore, because of the variation in the flexibility [6,8] or persistence lengths of these proteins [6,9,10,11]), which presumably cause azimuthal shift of the Tpm strands [5,6,7,8,9,10,11]. At a low Ca^2+^, troponin I interacts with F-actin [12], switching thin filaments off [7], which leads to spatial rearrangement and an increase in the persistence length of the actin filament [10,11]. At the same time, the persistence length of Tpm decreases [10,11] and restricts Tpm to a position close to the outer domains of actin (the blocked position) [7]. In this state of the thin filament (the “off” state) [7], the strong binding of myosin with actin is inhibited [2,5,7]. When Ca^2+^ binds to troponin C, some actin monomers change their conformation to the “switched-on” state, and the persistence length of the actin filament decreases [10,11]. At the same time, the persistence length of Tpm increases [10,11], and Tpm moves towards the inner domains of the actin [6,7,12], partly exposing the myosin-binding sites (closed position) [7]. When the myosin heads are strongly bound to the F-actin filament, the actin monomers are switched on, the persistence length of the actin filament decreases, and the persistence length of Tpm increases [10,11]. In this state (the “on” state), the Tpm strand completely exposes the binding sites of F-actin to myosin and initiates muscle contraction [7,13]. Recently, the amino acid residues that are involved in Tpm-actin interaction have been identified [14,15]. In addition, it was suggested that tropomyosin can bind to the myosin head, regulating the binding of the latter to actin [14]. Consequently, tropomyosin is a central link in the regulation of muscle contraction.

In skeletal muscle, there are three main Tpm isoforms, namely, α-, β-, and γ-Tpm, which are encoded by the TPM1, TPM2, and TPM3 genes, respectively [16]. All three isoforms exist either as homodimers or heterodimers [17,18]. Mutations in the Tpm genes give rise to a wide spectrum of clinically, histologically, and genetically variable neuromuscular and cardiac disorders. The numerous point mutations in the TPM2 and TPM3 genes were found in patients with congenital pathologies such as nemaline myopathy, cap-myopathy, distal arthrogryposis, congenital muscle fiber type disproportion (CFTD) and others, that are characterized by muscle weakness and hypotension (for reviews, see [18,19,20,21,22,23]). A number of Tpm mutations associated with congenital myopathies (K7X, K49X, K128E, R90P/C, R91G, R167C/G/H, K168E, and R245G/I) affect conserved residues with charged radicals along the entire length of the tropomyosin surface, that electrostatically interact with the Asp25 residue of actin monomers or are adjacent to such a residue [19,20]. Molecular dynamics simulations have predicted that a decrease in charge due to these substitutions and deletions can increase the distance between Tpm and the axis of the actin filament and change the position of Tpm when contacts with actin are broken [18]. One of these mutations, R90P in Tpm3.12, encoded by the TPM3 gene, was detected with hypotonia, feeding difficulties, motor delay, and scoliosis, requiring non-invasive ventilation while ambulant [23]. Muscle biopsies showed fiber type disproportion without other morphological changes in the skeletal muscle tissue.

CFTD is a rare genetic muscle disease. Severe progressive weakness and serious complications such as dysphagia and respiratory distress develop in about 25% of cases. Now, a diagnosis of CFTD is considered if type I fibers are at least 35–40% smaller than type II fibers, although previously a 12% disproportion was accepted. Fiber type disproportion is not specific and occurs in association with many other disorders or conditions. Therefore, the diagnosis of CFTD is controversial, and is suggested to be used when there are no other identifiable clinical and histological features. The molecular mechanisms underlying the development of this disease are unknown.

Here, we studied the effect of Arg for Pro residue substitution in the 90th position in the recombinant Tpm3.12 on the actin–myosin interaction at different simulated stages of the ATPase cycle (±Ca^2+^). Models of striated muscle fibers (so-called ghost fibers), where, because of the extraction of myosin and the regulatory proteins, actin comprised up to 80% of the total muscle protein, were used in this work. Spatial rearrangements of actin, myosin head (myosin subfragment-1, S1), and tropomyosin modified by fluorescent probes were studied using polarized fluorimetry, which is a highly applicable approach in this kind of research [8,24,25]. The results show that tropomyosin with the R90P substitution changes the proportion of switched-on and switched-off actin monomers, the balance of S1 strongly and weakly bound with F-actin, and the position of tropomyosin itself during the ATPase cycle. It is assumed that the R90P substitution induces conformational changes in Tpm, that fix Tpm strands near the open position, weaken the ability of troponin I to switch the actin monomers off, activate the weak binding of the myosin heads to F-actin at a low Ca^2+^, and induce the appearance of the strongly bound myosin heads during the ATPase cycle and under relaxing conditions. This may be the main cause for both the abnormally high Ca^2+^-sensitivity and muscle weakness. It was shown that the inhibitor of the ATPase activity of myosin BDM and the Ca^2+^-desensitizer W7 are able to weaken the effect of this mutation on myofilament’s sensitivity to the Ca^2+^ concentration.

## 2. Results and Discussion

### 2.1. The R90P Mutation in Tpm3.12 Produces Abnormally High Myofilament Ca^2+^-Sensitivity in Protein Solution

**Figure 1:**
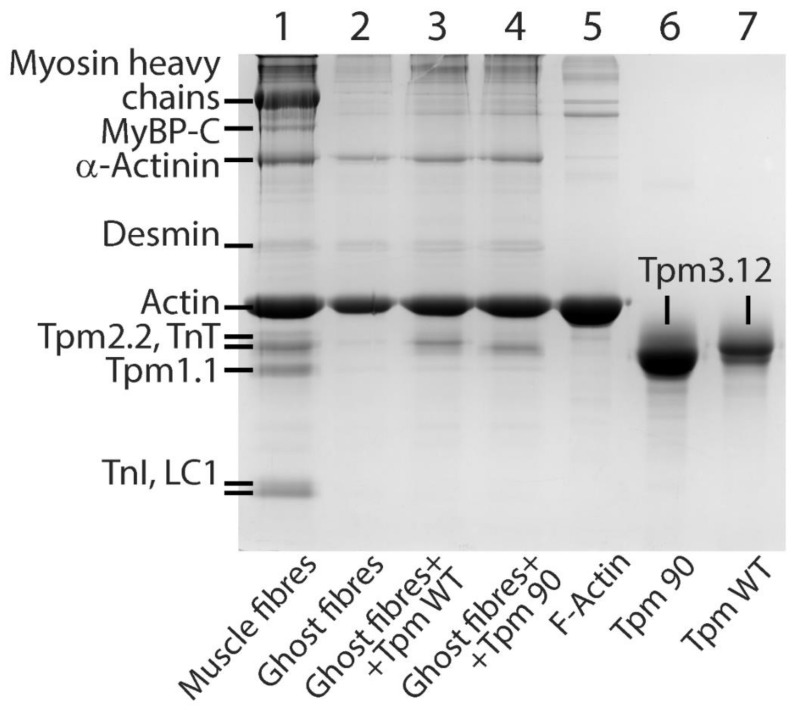
Composition of the muscle fibers (lane 1), ghost fibers (lane 2), and ghost fibers reconstituted with the wild-type (WT, lane 3) or R90P-mutant (lane 4) tropomyosins (Tpm). The unbound Tpm was removed by exposing the fibers to the washing solution for 15 min. Here, 8–10 fibers were used to prepare the probe loaded per gel. The preparations of F-actin, as well as the mutant and WT Tpms, were loaded on lanes 5, 6, and 7, respectively. According to the calculation using Image Lab 6.0, ghost fibers are composed of F-actin by 86.0 ± 0.4%. The ratio of the R90P-mutant Tpm bound to actin is 19 ± 2% lower than that of the WT-Tpm. Designations: Tpm1.1, Tpm2.2, and Tpm3.12—tropomyosin isoforms; MyBP-C—myosin-binding protein C; LC1—myosin light chain 1; TnT and TnI—troponin subunits.

We first evaluated the effect of the R90P substitution in Tpm3.12 on the Ca^2+^-sensitivity of reconstituted thin filaments. The filaments were assembled with the wild-type tropomyosin (WT-Tpm) or R90P-Tpm (Figure 1), and were used in the measurements of the actin-activated S1 ATPase activity at increasing Ca^2+^ concentrations (see Materials and Methods).

**Figure 2:**
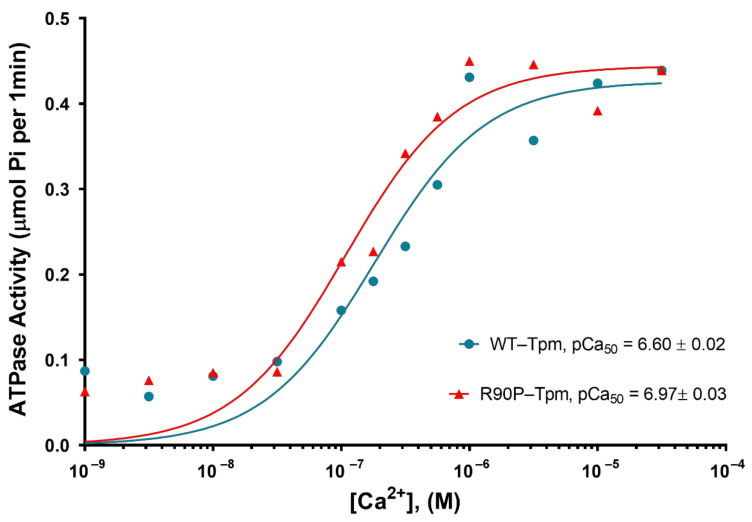
The effects of the increasing Ca^2+^ concentration on the actin-activated ATPase activity of S1. Ca^2+^ dependence was determined for fully regulated reconstituted thin filaments in the presence of WT-Tpm (blue circles) or R90P-Tpm (red triangles). The figure shows one of the typical graphs. The average values for pCa_50_ from 3–4 independent experiments are presented. Errors indicate ± SEM. Other conditions are as described in Materials and Methods.

As indicated by a leftward shift in the pCa–ATPase curve obtained in the presence of R90P-Tpm, the mutation increases the Ca^2+^-sensitivity of the thin filaments in the protein solution (Figure 2).

The midpoints of the curves (pCa_50_) were 6.97 ± 0.03 for the filaments containing the R90P-Tpm, and 6.60 ± 0.03 for those reconstituted with WT-Tpm (*p* < 0.01), showing an increase in the myofilament Ca^2+^-sensitivity. A slight increase or no significant difference in the actin-activated ATPase activity of S1 at a high Ca^2+^ was found in the presence of R90P-Tpm compared with WT-Tpm (Figure 2).

### 2.2. The R90P Mutation in Tpm3.12 Depresses the Ca^2+^-Dependent Tpm’s Movement on the Actin Filaments

In this work, we used a model system of thin filaments reconstituted in ghost muscle fibers using exogenous tropomyosin and troponin, decorated them with myosin subfragment-1, and mimicked several steps of the ATP hydrolysis cycle [8,9,10,11]. Prior to reconstitution, the ghost muscle fibers were completely devoid of tropomyosin, troponin, and myosin (see Materials and Methods; Figure 1). Exogenous tropomyosin (WT-Tpm or R90P-Tpm), troponin, and S1 were incorporated into the ghost fibers. Control experiments, including SDS–PAGE analysis (Figure 1), showed that the relative amount of the incorporated protein remained practically the same for each protein in all of the experiments.

In order to study the effect of the R90P replacement in Tpm3.12 on the behavior of Tpm and the impact of the myosin heads and actin on the movement of Tpm during the ATPase cycle, we used polarized fluorimetry [8,9,10,11,24,25]; the polarized fluorescence of the studied protein reflects the average structural state of the population of molecules of this protein [8,9,10,11]. The AM state of the actomyosin complex was simulated in the absence of nucleotides. Mg^2+^-adenosine diphosphate (MgADP) and Mg^2+^-adenosine triphosphate (MgATP) were used to mimic the AM^·ADP and AM**·ATP states, respectively [8,9,10,11,26].

**Figure 3:**
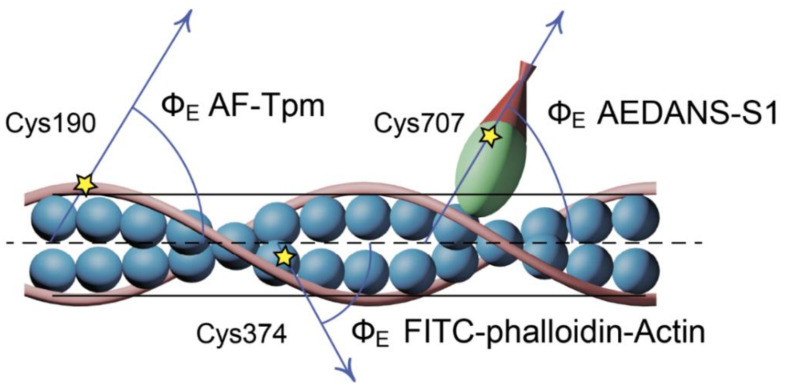
A schematic view of the localization of 5-IAF, FITC-phalloidin, and 1,5-IAEDANS in Tpm, F-actin (Actin), and myosin subfragment-1 (S1), respectively, in the ghost muscle fibers.

**Figure 4:**
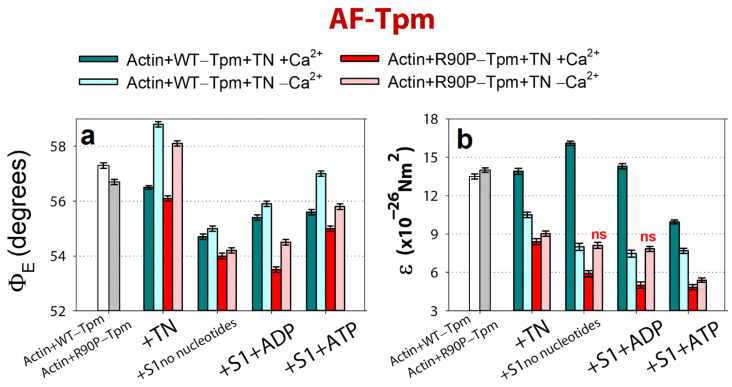
The effect of troponin (TN) and Ca^2+^ on the values of Φ_E_ (**a**) and ε (**b**) of the polarized fluorescence for 5-IAF linked with the WT-Tpm and R90P-Tpm, revealed in ghost fibers under conditions simulating the sequential steps of the actomyosin ATPase cycle. The values of Φ_E_ were corrected in order to take into account the changes in the conformation of Tpm strands. The first and second entries from the left in each panel present the data obtained in the absence of S1. The data represent the mean values for 5–7 fibers for each experiment (see Materials and Methods). The Φ_E_ and ε values in the absence and in the presence of nucleotides are significantly altered by TN and Ca^2+^ for both WT-Tpm and R90P-Tpm (*p* < 0.05). Mark “ns” indicates unreliable differences in the values of ε between the WT and mutant Tpms. Error bars indicate ± SEM. The values of N are close to zero.

Tropomyosin was labeled with a fluorescent probe 5-iodoacetamidofluorescein (5-IAF), which was covalently linked to the Cys190 residue [8,27] and allowed for determining the changes in the flexibility and spatial arrangement of the Tpm strands in the muscle fibers (Figure 3 and Figure 4).

The modification of Tpm by 5-IAF has been shown to have no significant effect on the functional properties of this protein [26], thus the observed changes in the conformational state of the labeled Tpm are likely to reflect those occurring during muscle contraction [8,9,10,11].

As shown by SDS–PAGE (Figure 1), the amount of the bound R90P-Tpm was 81 ± 2% of the amount found in the fibers reconstructed with the WT-Tpm, which presumably reflects the lower affinity of R90P-Tpm for actin. The incorporation of AF-WT-Tpm-TN or AF-R90P-Tpm-TN into the actin filaments of the ghost fibers (Figure 1) initiated polarized fluorescence [8,9,10,11]. The fluorescence intensity I_sum_ = (_⊥_I_⊥_ + _||_I_||_ + _||_I_⊥_ + _⊥_I_||_)/n, where n is the number of measurements, was 223 ± 15 and 246 ± 18 (*n* = 25) for the AF-WT-Tpm and AF-R90P-Tpm, respectively. This implies that in the ghost fibers, the fluorescence intensity of the mutant tropomyosin did not differ from that of the WT tropomyosin. Thus, the R90P alteration had no significant effect on the number of tropomyosin molecules associated with the thin filaments in the muscle fibers.

As illustrated in Figure 4a,b, the binding of TN to the actin-AF-WT-Tpm complex at a high Ca^2+^ results in a decrease in the values of Φ_E_ from 56.8 to 56.3°, and an increase in the values of ε from 13.5 × 10^−26^ N∙m^2^ to 13.9 × 10^−26^ N∙m^2^, *p* < 0.05. In our previous works [8,9,10,11] and here, we relied on the observation done in the electron microscopy studies [5,6,7] about the azimuthal shifting of Tpm strands relative to the outer and inner actin domains in different regulatory states of the thin filament. A decrease in the Φ_E_ value for 5-IAF-labeled Tpm has been associated with the transition between the regulatory states characterized by the shifting of Tpm to the inner domains of actin (towards the closed position). On the contrary, an increase in the Φ_E_ value is considered to indicate a shifting of Tpm to the outer domains of actin subunits (towards the blocked position) [8,9,10,11].

**Figure 5:**
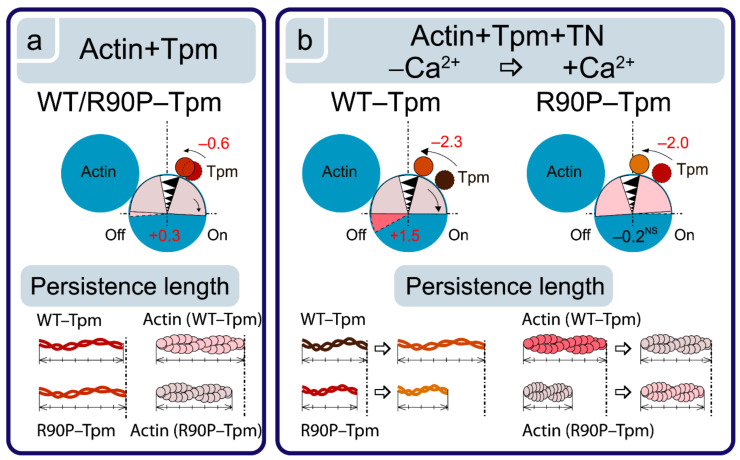
Schematic explanation of the changes in the tropomyosin (Tpm) position, angular orientation of the actin monomer, and persistence lengths of the Tpm strands and actin filaments due to the R90P substitution in Tpm. The information was obtained from the calculation of the value for Φ_E_ (the angle of orientation of the emission dipoles of the fluorescent probes bound to Tpm or actin) and of the value for ε (the bending stiffness; shown here as the alterations in the persistence length of the Tpm strands and actin filament drawn to scale). Ghost fibers were composed of actin and Tpm (**a**) or of actin, Tpm, and TN (**b**). The Tpm position and actin monomers orientation are affected by the R90P mutation and are typical for the shift of Tpm to the inner domains of actin, and for the switching of actin monomers on (clockwise rotation). The mutation decreases the persistence lengths of Tpm and actin. The R90P replacement reverses the changes in the persistence lengths of Tpm and actin that occur in response to the rising Ca^2+^ concentration (**b**). Designations: the changes in Φ_E_ between the states at a high and a low Ca^2+^ are shown by numbers, and the direction of the rearrangements is depicted by arrows. The non-significant change in Φ_E_ is marked with superscript “NS”. Different localization of Tpm and conformational states of actin and respective persistence lengths are depicted by different colors. Red for numbers is used when the changes are typical for the transition of the complex to the activated state.

An essential point is the correlation between the changes in the values of Φ_E_ and ε for 5-IAF-labeled Tpm (Figure 4b). Here, the ε value is a bending stiffness of Tpm (see Materials and Methods). An increase in the value for ε was shown to correlate with the elongation of Tpm, while a decrease in this value is correlated with its shortening. It is to be noted that larger changes in ε are correlated with larger alterations in Φ_E_ (Figure 4b and Figure 5b). Thus, the changes in the values for Φ_E_ and ε contain information on how far and in which direction the Tpm strands shift [10,11]. The decrease in the Φ_E_ value observed with the WT-Tpm and at binding of TN to F-actin at a high Ca^2+^ (Figure 4a,b), could be explained by a relocation of the Tpm strand towards the inner domains of actin, to the open position, and by an increase in the persistence length of Tpm (Figure 5b). Similar azimuthal shift of the Tpm strands at the binding of TN to F-actin at a high Ca^2+^ were observed earlier [3,4,5,6,7].

At a low Ca^2+^, the value for the angle Φ_E_ increased by 2.0° and for ε decreased by 2.0 × 10^−26^ N∙m^2^ for the actin-AF-WT-Tpm complex compared with the values observed at a high Ca^2+^. It seems plausible that the decrease in the ε value for AF-WT-Tpm reflects a decrease in the persistence length of Tpm [10,11], which would make it to move towards the outer domains of actin at a low Ca^2+^ [5,6,7]. Thus, at a high Ca^2+^, the WT-Tpm shifted in the direction of the inner domains of actin (to the closed position, Figure 5b). On the contrary, at a low Ca^2+^, WT-Tpm shifted towards the outer domains of actin (Figure 5b, towards the blocked position, [5,6,7]).

**Figure 6:**
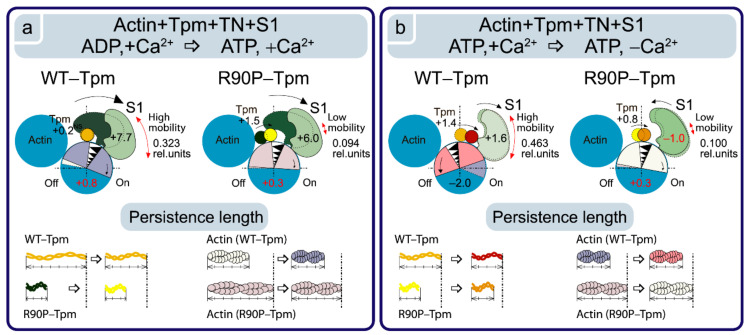
Schematic view of the alterations in the Tpm position, conformational rearrangements in actin and S1, and the changes in the persistence lengths of the Tpm strands and actin filaments due to the R90P substitution in Tpm, as follows from the polarized fluorimetry data. The changes in the value for Φ_E_ are interpreted as the shift of Tpm relative to the inner and outer domains of actin, the rotation of actin monomers in the actin filament and the azimuthal tilt of the myosin motor domain. The left and right panels show the transition of the Actin-Tpm-TN-S1 complex from the state in the presence of ADP at a high Ca^2+^ to the state in the presence of ATP at a high Ca^2+^ (**a**), and further to the state at a low Ca^2+^ concentration (**b**). The mutant Tpm strands have a lower persistence length and are shifted towards the inner domains of actin as compared with WT-Tpm. The persistence length changes are drawn to scale. The mutation increases the persistence length of actin, inhibits the actin monomer rotation, and causes the switching of actin monomers off at the transition from the state in the presence of ADP to that in the presence of ATP and the switching of actin monomers on at a low Ca^2+^. The abnormal position of the mutant Tpm induces the appearance of the myosin heads strongly bound to actin under relaxing conditions. The mobility of the myosin heads is decreased in the presence of the mutant Tpm, as follows from the calculation of the number of the disordered probes (parameter N). Designations are the same as in Figure 5. Different conformational states of the myosin head are depicted by different shades of green.

The similar Ca^2+^-dependence was observed in the presence of the myosin heads (S1) at mimicking various states of the ATPase cycle. The values for Φ_E_ and ε were always lower and higher, respectively, at a high Ca^2+^, and vice versa, higher and lower at a low Ca^2+^, respectively. The highest values for Φ_E_ and the lowest values for ε were observed for AF-WT-Tpm in the presence of ATP at a low Ca^2+^, while the Φ_E_ values were the lowest and ε values were the highest at a high Ca^2+^ in the absence of nucleotides (Figure 4a,b and Figure 6). Thus, upon transition from the relaxation to the rigor state, the Tpm strands shift from the blocked to the open position (the values of Φ_E_ become smaller and the persistence length of the Tpm strands becomes larger (Figure 4a,b and Figure 6). A similar correlation was observed previously [10,11].

It is known that the Arg for Pro residue replacement at position 90 in Tpm3.12 leads to substantial structural changes, sharply reducing the thermal stability of the entire Tpm molecule, especially its N-terminal part. Most likely, this is due to the fact that the introduction of a proline residue by the R90P mutation strongly disrupts the structure of the α-helix and, accordingly, the double α-helix in the N-terminal part of γγ-Tpm, where residue 90 is located; these changes are transmitted to the C-terminal region of the molecule, reducing its thermal stability [28]. Moreover, this mutation also weakens the stability of the Actin-R90P-Tpm complex and depresses the maximal sliding velocity of the reconstituted thin filaments at a saturating Ca^2+^ concentration [28].

In our work, we observed that the R90P mutation results in a decrease in the values of Φ_E_ from 57.3 to 56.7°, and a slight increase in the values of ε for the Actin-AF-R90P-Tpm complex (Figure 4a,b), which could be explained by a relocation of Tpm towards the inner domains of actin. We postulated that the R90P mutation “fixes” the Tpm strands closer to the open position during the ATPase cycle (Figure 4a,b). Indeed, when AF-R90P-Tpm was bound to Actin-S1 at a high Ca^2+^, at mimicking different states of S1, the values of Φ_E_ were distinctly smaller than for AF-WT-Tpm under the same conditions (Figure 4a and Figure 6), showing that the mutant Tpm is located closer to the inner domains of actin than the wild-type tropomyosin during the ATPase cycle.

According to Figure 4a, when mimicking the “off” state, the value of Φ_E_ was essentially smaller for the R90P-Tpm than for WT-Tpm, which agrees well with the putative movement of the mutant Tpm towards the inner domains of actin (Figure 6). The similar effect was observed when mimicking various states of the ATPase cycle (Figure 4a,b and Figure 6). Thus, our assumption seems to be correct, and the R90P mutation shifts the Tpm strands towards the open position both at a low and a high Ca^2+^, reducing the ability of Tpm to the Ca^2+^-dependent movements (making the Tpm stands to “freeze” near the open position; Figure 6).

It should be noted that the R90P alteration dramatically increases the flexibility of Tpm when mimicking the relaxation of a muscle fiber (Figure 4b). This, together with an abnormal movement of the Tpm to the inner domains of actin, can contribute to the emergence of a marked number of myosin heads bound strongly to F-actin. In addition, localization of the mutant Tpm in proximity to the open position during the ATPase cycle may suppress the ability of troponin to switch actin monomers off at a low Ca^2+^. Indeed, these changes were observed (see below).

### 2.3. The R90P Mutation Inhibits Troponin’s Ability to Switch Actin Monomers off at a Low Ca^2+^

In this work, FITC-phalloidin was bound to F-actin in the region of the actin groove [29] (Figure 3), which initiated polarized fluorescence and allowed for determining the changes in flexibility and spatial arrangement of the actin subunits in the thin filaments (see Materials and Methods) at various stages of muscle contraction [8,9,10,11].

It is known that phalloidin increases the stiffness of the actin filament [29]. The effect of phalloidin on the actin-activated ATPase activity of the skeletal muscle myosin was studied by Dancker and co-workers on isolated actomyosin [30], and by Bukatina and Fuchs on myofibrils [31]. The first group of researchers found no effect of phalloidin, while the second reported a Ca^2+^-dependent increase in ATPase activity. With 50 μM phalloidin added, a maximal increase of 25% was observed at pCa 8, whereas at pCa 4, there was no increase in the ATPase activity [31]. However, our control experiments revealed no effect of FITC-phalloidin on the ATPase activity of S1 [32,33]. Based on these facts, we concluded that the effect of FITC-phalloidin on the contractile apparatus of striated muscle was negligibly small [34].

**Figure 7:**
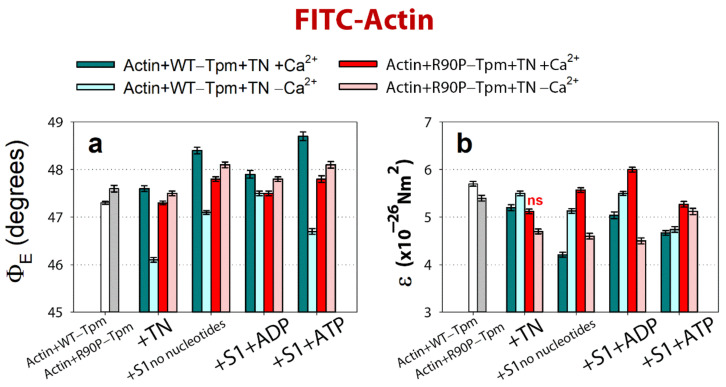
The effect of troponin and Ca^2+^ on the values of Φ_E_ (**a**) and ε (**b**) of the polarized fluorescence for FITC-phalloidin-Actin, in the presence of the WT-Tpm or R90P-Tpm under conditions simulating the sequential steps of the myosin ATPase cycle. The first and second entries from the left in each panel present the data obtained in the absence of S1. Calculations of Φ_E_ and ε values, preparation of the fibers, their composition, and the conditions of the experiments are described in Materials and Methods. The data represent the mean values for 8–10 fibers for each experimental condition. Both in the absence and in the presence of nucleotides, the Φ_E_ and ε values for the WT-Tpm and R90P-Tpm are significantly altered by troponin and Ca^2+^ (*p* < 0.05). Index “ns” marks unreliable difference in the value for ε between the WT and mutant tropomyosins. Error bars indicate ± SEM. The values of N are close to 0.1.

At a high Ca^2+^, the binding of TN to the FITC-actin-WT-Tpm complex induced an increase in the value for Φ_E_ by 0.3°, *p* < 0.05, and a decrease in the value for ε by 0.5 × 10^−26^ N∙m^2^, whereas at a low Ca^2+^, the values for Φ_E_ and ε decreased by 1.2° and 0.2 × 10^−26^ N∙m^2^, *p* < 0.05 (Figure 7a,b), respectively. According to our earlier published data, alterations in the Φ_E_ and ε values for FITC-actin may be interpreted as a result of conformational changes (global and/or local), accompanied by switching of the actin monomers on and off, respectively, which is associated with an enhancement or a reduction in the ability of F-actin [8,9,10,11] to activate the myosin ATPase [2]. The value of ε for FITC-actin-WT-Tpm-TN was lower at a high Ca^2+^ than at a low Ca^2+^ (Figure 7b). An increase and a decrease in the flexibility of the thin filaments was correlated with a reduction and rise, respectively, in the persistence length of F-actin [8,9,10,11] (Figure 5b and Figure 7b). At a low Ca^2+^, TN switches the actin monomers off and induces an increase in the persistence length of F-actin (Figure 5b and Figure 7b). It is known that at a low Ca^2+^, troponin I can bind to F-actin [34,35] and induce conformational changes in F-actin that lead apparently to a raised number of the switched-off actin subunits [8,9,10,11]. It was postulated that in this case, monomers are turned to the filament axis (rotated counterclockwise, Figure 5), which hindered the binding of the myosin heads to actin, as opposed to the orientation of the actin monomers in the switched-on state [10,11]. A similar increase and decrease in the persistence length of the thin filaments was observed by Isambert and coworkers [36] at lowering and rising Ca^2+^, respectively.

The R90P mutation in Tpm significantly affected the ability of troponin to switch actin monomers on and off in the thin filaments. Indeed, when the R90P-Tpm-TN complex was bound to FITC-actin, at a high Ca^2+^, the values for Φ_E_ decreased from 47.6 to 47.3° and the values for ε fell from 5.7 × 10^−26^ N∙m^2^ to 5.1 × 10^−26^ N∙m^2^, *p* < 0.05, respectively, whereas for the WT-Tpm, on the contrary, a decrease in these values was observed under the same conditions (Figure 7a,b). The decrease in the values for Φ_E_ and ε may be interpreted as pointing out to a reduction in the fraction of the switched-on actin monomers (an increase in this fraction is typical for the transition of the thin filament to the on state at a high Ca^2+^ [7]). Thus, at a high Ca^2+^, the R90P mutation decreases the proportion of switched-on actin monomers in the thin filament, despite the fact that the R90P-Tpm is shifted to the inner domains of the actin (Figure 4a,b). The disconnected work of tropomyosin and actin in the thin filaments may be one of the reasons for the absence of a noticeable effect of this mutation on the ATPase activity of S1 at a high Ca^2+^ (Figure 2). A violation of the coordinated work of these proteins in the thin filaments was found earlier for other mutant Tpms [9,33].

At a low Ca^2+^, the values for FITC-actin-R90P-Tpm-TN were higher by 1.4° (47.5° vs. 46.1°) for Φ_E_ and lower by 0.9 × 10^−26^ N∙m^2^ (5.6 vs. 4.7) for ε, than the correspondent values for WT-Tpm (Figure 4a,b). This implies that at a low Ca^2+^, the R90P-Tpm caused a turn of actin monomers from the filament axis (a clockwise rotation, Figure 5b) and a decrease in the persistence length of F-actin in the thin filaments, with the actin monomers being predominantly in the switched-on state [8,9,10,11]. Consequently, R90P-Tpm reduced the ability of TN to switch actin monomers off in the thin filaments (Figure 7a,b).

Our data indicate that the R90P mutation alters the ability of TN to shift Tpm in a Ca^2+^-dependent manner (Figure 4a,b). Indeed, at a low Ca^2+^, instead of shifting Tpm towards the outer domains of actin to the blocked position (where Tpm would prevent myosin heads to bind strongly with F-actin), TN moves the mutant tropomyosin towards the inner domains of actin, towards the open position (Figure 4a,b). As in the closed position, where Tpm strands allow for a strong binding of the myosin heads to F-actin [7], it is suggested that the shifting of the R90P-Tpm towards the open position at a low Ca^2+^ can contribute to the increase in Ca^2+^-sensitivity observed here (Figure 2). There is evidence that at a low Ca^2+^, troponin I bridges the adjacent actin subunits across the filament and the residues 157–163 of troponin I interact with the residue 146 of Tpm on the opposite actin helix [34]. This interaction induces Tpm movement towards the blocking position [35]. The R90P mutation inhibits this movement (Figure 4a). As residues 90 and 146 are far from each other, the R90P substitution seems to induce such conformational changes in Tpm that capture the regions located near the site of the Tpm interaction with troponin, that is, the R90P mutation is likely to cause a global disturbance of the tropomyosin molecule [28], which inhibits the interaction of troponin I with Tpm.

Thus, the R90P mutation in Tpm inhibits the ability of TN to switch actin monomers off (Figure 7a,b), and makes TN unable to move Tpm strands towards the blocked position at a low Ca^2+^ (Figure 4a,b). Being located near the open position, R90P-Tpm does not block the binding of myosin to actin at a low Ca^2+^ [7]. Consequently, the abnormal position of the mutant Tpm and the inability of TN to switch the actin monomers off may be the main cause for both the abnormally high Ca^2+^-sensitivity (Figure 2) and muscle weakness (see below).

### 2.4. The R90P Mutation Allows the Strong Binding of the Myosin Heads to F-Actin at a Low Ca^2+^

In this work, myosin subfragment-1 was specifically modified by N-(iodoacetaminoethyl)-1-naphthyl-amine-5-sulfonic acid (1,5-IAEDANS) at its Cys707 residue, which initiated polarized fluorescence and allowed for determining the changes in the flexibility and spatial arrangement of the myosin heads in the muscle fibers [8,9,10,11].

Modification of Cys707 with a fluorescent probe may affect some aspects of myosin’s behavior, but remains a valid means of gaining information on the actin–myosin interaction. The labeling of Cys707 can reduce the ATPase activity of myosin and the sliding velocities of actin over myosin [37,38]. Furthermore, it has been shown that the modification diminishes the rotation of the converter region of the myosin head that takes place during the ATPase cycle [39]. However, myosin heads modified with fluorescent probes retain nucleotide sensitivity. In particular, Cys707 modification by 1,5-IAEDANS has no effect on the strong binding (in the absence of nucleotide or in the presence of MgADP) and the weak binding (in the presence of MgATP) of S1 to actin [38]. In our control experiments, we also did not find any essential effect of the modification on the strong binding (in the absence of nucleotides or in the presence of MgADP) or on the weak binding (in the presence of MgATP) of S1 to actin [40]. Thus, within the experimental design used in this work, AEDANS-S1 may be used for studying actin–myosin interaction during the ATPase cycle [8]. We used modified myosin heads in order to determine whether mutant Tpm can affect the strong and weak binding of myosin heads to F-actin. The change in binding was assessed based on the alterations in the orientation and mobility of the myosin heads [8,9,10,11].

**Figure 8:**
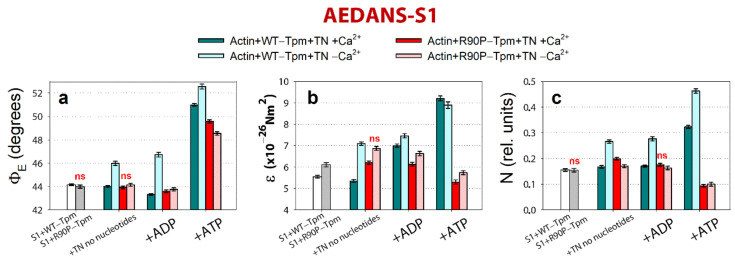
The effect of troponin and Ca^2+^ on the values of Φ_E_ (**a**), ε (**b**), and N (**c**) of the polarized fluorescence of N-(iodoacetaminoethyl)-1-naphthyl-amine-5-sulfonic acid (1,5-IAEDANS) bound to S1 (AEDANS-S1), in the presence of WT-Tpm or R90P-Tpm, revealed in ghost fibers under conditions simulating the sequential steps of the actomyosin ATPase cycle. The data represent the mean values for 5–7 fibers for each experimental condition. The Φ_E_, ε, and N values in the absence and in the presence of TN, Ca^2+^, and nucleotides are significantly altered by the R90P substitution in Tpm (*p* < 0.05). Statistically insignificant differences in the values of Φ_E_, ε, and N between the WT and mutant Tpms are marked by the symbol “ns”. Error bars indicate ± SEM.

According to Figure 8, for the actin-WT-Tpm-AEDANS-S1 complex, the values for the angle Φ_E_ between the fiber axis and the emission dipole of the probe, the bending stiffness (ε), and the relative number of the disordered fluorophores (N) were found to be equal to 44.2°, 5.55 × 10^−26^ N∙m^2^, and 0.156 rel. unit, respectively. This indicated that the probes were highly oriented and the myosin heads were bound strongly to F-actin [8,9,10,11]. As the fluorescent probe was rigidly bound to S1, it was assumed that the parameter ε contains information about the binding stiffness of the F-actin filaments in the region of localization of the myosin heads, whereas parameter N allows for estimating the flexibility of the attachment of the myosin heads to F-actin [11]. The binding stiffness of actin filaments, which was determined using the polarized fluorescence from FITC-phalloidin (Figure 7b), did not differ much from that for F-actin in the areas of the localization of myosin heads in all of the experimental conditions (Figure 8b). This observation demonstrates the possibility of propagation for the changes in the conformation of actin monomers along the thin filament. The transition of the signal along the thin filament was shown earlier by Barua et al. [14].

The binding of TN to the actin-WT-Tpm-AEDANS-S1 complex induced the following change in the values of Φ_E_, ε, and N: at a high Ca^2+^, they slightly decreased by 0.2°, 0.2 × 10^−26^ N∙m^2^, and 0.02 rel. units (*p* < 0.05), respectively (Figure 8). Such changes in the values of these parameters can be interpreted as showing an increase in the amount of myosin heads strongly bound to F-actin in the ghost muscle fibers [8,9,10,11,41]. On the contrary, at a low Ca^2+^, these parameters essentially increased by 2.0°, 1.5 × 10^−26^ N∙m^2^, and 0.11 rel. units (*p* < 0.05), respectively (Figure 8). Thus, the WT-Tpm-TN complex was able to facilitate (at a high Ca^2+^) and inhibit (at a low Ca^2+^) the strong binding of the myosin heads to the thin filaments.

A similar pattern of changes in the parameters at a high Ca^2+^ was also observed upon mimicking the AM^·ADP and AM**ATP stages of the ATPase cycle (Figure 8). The presence of MgADP induced the strong binding of the myosin heads to F-actin; the values for Φ_E_ were smaller and for ε and N were higher than in the absence of MgADP. In the presence of MgATP, a decrease in the amount of myosin heads strongly bound to actin was observed (the values for Φ_E_, ε, and N were higher than in the presence of MgADP, Figure 8). At a low Ca^2+^, the amount of myosin heads strongly bound to F-actin extremely decreased (in the presence of MgADP the values for Φ_E_, ε and N were higher by 3.4°, 0.46 × 10^−26^ N∙m^2^, and 0.11 rel. units, respectively, and in the presence of MgATP the Φ_E_ and N values were higher by 1.57° and 0.14 rel. units, respectively, than the corresponding values obtained at a high Ca^2+^, Figure 8). Consequently, upon mimicking the strong-binding states of the ATPase cycle, the WT-Tpm located close to the open position (Figure 4), the amount of the switched-on actin monomers (Figure 7) and strongly bound myosin heads (Figure 8) were higher than when mimicking the weak-binding state. At a low Ca^2+^, WT-Tpm moves towards the blocked position (Figure 4a), the amount of switched-on actin monomers (Figure 7) and the myosin heads strongly bound to actin decrease (Figure 8). The R90P mutation altered this picture.

As opposed to the actin-WT-Tpm-TN-AEDANS-S1 complex, the complex where the WT-Tpm was replaced by R90P-Tpm showed an increase in the number of myosin heads strongly bound to F-actin at a low Ca^2+^ (in the absence of a nucleotide, the Φ_E_, ε, and N values were lower for R90P-Tpm, than for WT-Tpm by 2.0°, 0.23 × 10^−26^ N∙m^2^, and 0.095 rel. units, respectively, *p* < 0.05, Figure 8).

A similar increase in the amount of the myosin heads strongly bound to actin at a low Ca^2+^ was also observed in the presence of MgADP and MgATP (at mimicking the AM^·ADP and AM**ATP states). In the presence of MgADP at a low Ca^2+^, the values for Φ_E_, ε, and N were reduced by 3.0°, 0.83 × 10^−26^ N∙m^2^, and 0.114 rel. units, respectively, in the presence of the mutation. In the presence of MgATP, the mutation decreased the value for Φ_E_ by 4.0° and had an impressive effect on the bending stiffness of F-actin at the site of localization of the myosin head and on the flexibility of myosin head attachment to F-actin (Figure 8c). Indeed, at a low Ca^2+^ (at mimicking relaxation), the values for ε and N decreased by 39% and 79%, respectively, which demonstrated—instead of anticipated increase in the rigidity of F-actin and flexibility of myosin attachment to actin typical for relaxation—the raised flexibility of F-actin and the rigidity of the bonds between the myosin heads and actin. It is noteworthy that the binding stiffness was 5.74 × 10^−26^ N∙m^2^, which is typical for the AM state in the presence of WT-Tpm at a high Ca^2+^, and the flexibility of the attachment of the myosin heads to F-actin at mimicking relaxation was much lower for the R90P-Tpm than for the WT-Tpm (the value for N was 0.10 rel. units for the R90P-Tpm vs. 0.463 rel. units for WT-Tpm, Figure 8c), showing “fixation” of the myosin heads on the thin filament. Such changes in these parameters can be interpreted as the formation of the so-called rigor-like myosin heads in the muscle fibers [10,11]. The appearance of the rigor-like cross-bridges can cause not only serious violations of the relaxation of the muscle fiber, but also bring about disorganization of the thin and thick filaments. The appearance of these cross-bridges can contribute to the development of hypotonia and motor delay in infancy and respiratory failure in early teens, which was observed in a patient with the mutant R90P-Tpm [19].

Thus, the R90P mutation in Tpm is able to facilitate the strong binding of the myosin heads to F-actin at a low Ca^2+^ when mimicking different stages of the ATPase cycle. This may be one of the reasons for the high Ca^2+^-sensitivity in the protein solution (Figure 2).

At a high Ca^2+^, the R90P substitution slightly decreases or does not change the amount of the strongly bound myosin heads (the Φ_E_ value does not change when mimicking the AM state and increases by 0.2° (*p* < 0.05) when mimicking the AM^·ADP state, Figure 8a). Upon mimicking the AM**ATP state, parameters Φ_E_, ε, and N essentially decrease by 1.43°, 4.61 × 10^−26^ N∙m^2^, and 0.281 rel. units, respectively (*p* < 0.05, Figure 8). Therefore, the amplitude of changes in the values for Φ_E_ upon the transition of the myosin heads from the weak to the strong binding with F-actin during the ATPase cycle (between the weak binding in the presence of MgATP and the strong binding in the absence of the nucleotides at a high Ca^2+^) was 6.0° for the R90P-Tpm (Figure 8a), which was smaller than the amplitude observed for the WT-Tpm (7.7°). It is suggested that the R90P-Tpm can inhibit the efficiency of the cross-bridge work [42].

It is known that the substitution of positively charged Arg residue with neutral hydrophobic Pro residue that disrupts the coiled-coil structure dramatically destabilizes not only the N-terminal part of the Tpm molecule where it is located, but also its C-terminal part [28]. There is evidence that at a low Ca^2+^, troponin I bridges the adjacent actin subunits across the filament and the residues 157–163 of troponin I interact with the residue 146 of Tpm on the opposite actin helix [35]. As residues 90 and 146 are far from each other, the R90P substitution can block the interaction of the residue 146 to Tpm. Therefore, the R90P mutation can alter the binding of tropomyosin to troponin I. In this work, we observed that the mutant Tpm was shifted towards the open position upon mimicking all stages of the ATPase cycle at a high and low Ca^2+^ (Figure 4a). We suggest that an alteration in the binding the Tpm to troponin I could be the reason for the violated ability of troponin to switch the thin filaments off. This could lead to an increase in the Ca^2+^-sensitivity, and could induce the appearance of the rigor-like myosin heads, which were strongly bound to F-actin when mimicking relaxation in muscle fibers (Figure 8).

The ghost fibers are likely to also contain the giant protein nebulin, which extends from the Z-disk and may act as a ruler to determine thin filament length. It may also have some regulatory action on actomyosin. Nebulin is not soluble at physiological ionic strength; its extraction from the skeletal fibers requires the use of 1M urea, and its further separation from actin requires an increase in the concentration of urea to 4 M [43]. In our experiments, we did not use urea (see Material and Methods) and thus nebulin was not extracted from our ghost fiber preparations. We note that our two approaches for determining the functional effect of the R90P mutation—actin-activated myosin S1 ATPases carried out in the absence of nebulin, and polarized fluorescence measurements in ghost fibers containing nebulin—showed good agreement. Therefore, we believe that nebulin has no significant impact on the effect of the R90P mutation.

Similar rigor-like myosin heads as in the presence of the R90P-mutant Tpm were found by us when mimicking relaxation in the muscle fibers containing the other mutant isoforms of Tpm: E139X in β-Tpm [9], R91G in β-Tpm [44], R168G in α-Tpm [26], A155T [45], and E173A [11] in γ-Tpm. These mutations were associated with cap-myopathy, congenital muscle fiber type disproportion, and arthrogryposis, and were accompanied by contractures and disorganization in muscle fiber [19,22,23].

The damaging effects of the R90P mutation can be slightly restored by BDM and W7 (see below).

### 2.5. The Inhibitor of Myosin ATPase Activity, BDM, and the Ca^2+^-Desensitizer, W7, Are Able to Weaken the Ca^2+^-Sensitivity of Myofilaments Containing R90P-Tpm in Protein Solution

It was shown that the perturbation of the tropomyosin molecule caused by the substitution of Arg for Pro residue in the 90th position [28] causes conformational changes in Tpm that make the mutant Tpm to be located to near the open position during the ATPase cycle (Figure 4a,b). In addition, the troponin–tropomyosin complex loses both its ability to switch actin monomers off (Figure 7a,b) and to weaken the binding of the myosin heads to F-actin at a low Ca^2+^ (Figure 8a,c). As the regulation of muscle contraction is carried out by the concert interdependent conformational rearrangements of all proteins involved in this process, a change in the state of any of them will cause a change in the state of the entire system [9,10,11]. It has been known that the myosin heads bound strongly with F-actin and troponin at a high Ca^2+^ shift tropomyosin towards the open position [5,6,7], therefore it is possible to suggest that the suppression of the ability of the myosin heads to bind strongly with F-actin and the reduction of the Ca^2+^-sensitivity of troponin can bring about the mutant Tpm for normal functioning in the ATPase cycle.

Here, for depressing the strong binding of the myosin heads to F-actin, we used 2,3-butandione 2-monoxime (BDM). This reagent was first identified as an inhibitor of skeletal muscle tension [46,47,48,49], affected directly the muscle myosin [48], increasing the equilibrium constant for ATP hydrolysis and inhibiting the rate of phosphate release, thus stabilizing the M-ADP-Pi intermediate state [50,51].

**Figure 9:**
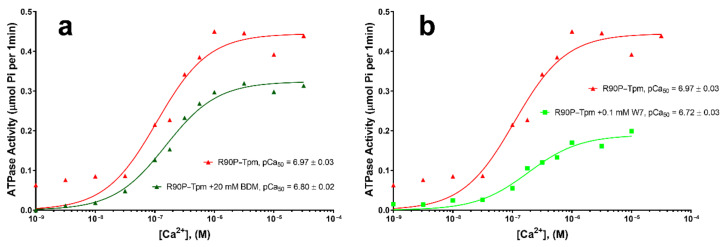
The effect of 20 mM BDM (**a**) and 100 µM W7 (**b**) on the sensitivity of the thin filaments containing the R90P mutation in γ-Tpm to activating Ca^2+^ concentrations. Ca^2+^-dependence is determined for fully regulated reconstituted thin filaments. The actin-S1 ATPase is measured in the presence of R90P-Tpm and 20 mM BDM or 0.1 mM W7 at 25 °C. Each panel shows one of the typical graphs. pCa values are calculated from the data averaged from three experiments. Errors indicate ± SEM.

Both BDM (20 mM) and W7 (0.1 mM) reduced the Ca^2+^-sensitivity of the thin filaments (Figure 9a,b). Here, 20 mM BDM shifted the midpoint of the curve (pCa_50_) to 6.80 ± 0.03 for the filaments containing the R90P-Tpm (Figure 9a), which may be associated with the movement of the Tpm strands towards the blocked position. Because a decrease in myofilament Ca^2+^-sensitivity in the presence of BDM may be caused by both the inhibition of the Tpm movement towards the open position at a low Ca^2+^ and the rehabilitation of troponin’s work, it is possible to postulate that BDM can restore the Ca^2+^-dependent regulation of the ATPase cycle in the protein solution.

It has been known that W7 (n-(6-aminohexyl) 5-chloro-1-napthalenesulfonamide) binds specifically with a high affinity to troponin C, but does not interact with actin, myosin, or Tpm [52], therefore it can be used as specific inhibitor of calcium activation in skinned fibers from skeletal muscles [52,53]. In addition, it was shown earlier that the desensitizer W7 can correct hyper-calcium-sensitivity of sarcomeres [54].

Here, we tried to use W7 to reduce the disruption of the actin–myosin interaction during the ATPase cycle in the ghost muscle fibers caused by the R90P mutation in Tpm. The binding of 0.1 mM of W7 shifted the midpoint of the curve (pCa_50_) to 6.72 ± 0.03 for the filaments containing the R90P-Tpm (Figure 9b), which demonstrates a shift of Tpm strands towards the blocked position (see below). This means that W7 can restore the Ca^2+^-dependent movement of the mutant Tpm during the ATPase cycle in the protein solution.

### 2.6. BDM and W7 Are Able to Weaken the Damaging Effect of the R90P Mutation on the Regulation of Muscle Contractility

We used polarized fluorimetry to determine the effect of BDM and W7 on the critical conformational changes in the myosin heads, F-actin, and tropomyosin of the thin filaments, caused by the R90P mutation in Tpm3.12.

**Figure 10:**
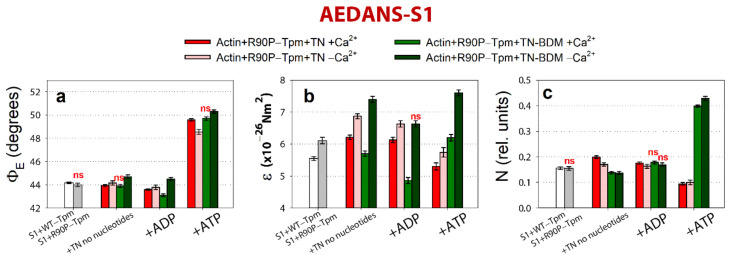
The effect of troponin and Ca^2+^ on the values of Φ_E_ (**a**), ε (**b**), and N (**c**) of the polarized fluorescence of 1,5-IAEDANS bound to S1 (AEDANS-S1), in the absence and in the presence of 50 mM BDM, revealed in ghost fibers containing R90P-Tpm under conditions simulating the sequential steps of the actomyosin ATPase cycle. The experimental conditions and designations are described in Materials and Methods. The data represent the mean values for 5–7 fibers for each experimental condition. The Φ_E_, ε, and N values in the absence of TN and in the presence of Ca^2+^ and nucleotides are significantly altered by BDM (*p* < 0.05). The index “ns” indicates statistically insignificant differences in the values of Φ_E_, ε, and N between the mutant tropomyosin in the absence or presence of BDM. Error bars indicate ± SEM.

As shown in Figure 10, the binding of 50 mM BDM to the actin-R90P-Tpm-TN-AEDANS-S1 complex induces a decrease in the values for Φ_E_, ε, and N at a high Ca^2+^ when mimicking the AM and AM^·ADP stages of the ATPase cycle.

On the contrary, at a low Ca^2+^, these values sharply increased, indicating a disorientation of the myosin heads, a weakening in their binding to actin, and a noticeable increase in the persistence length of actin filaments in thin filaments, i.e., changes typical for the contractile system at a low Ca^2+^ (see Figure 8). These changes are especially clearly seen in the persistence length of the R90P in the presence of MgATP and BDM. The values of N increased by 59% (Figure 10c), showing that in the presence of BDM, the flexibility of actin-bound myosin heads in the muscle fibers containing the mutant tropomyosin becomes the same as in the fibers containing the wild-type tropomyosin. It is known that BDM binds to myosin and fixes the myosin head in the AM-ADP-Pi intermediate state, which inhibits the release of phosphate, and this is possibly one of the reasons for the decrease in the ATPase activity of actomyosin in the presence of BDM (Figure 9a). Perhaps it is the inhibition of the phosphate release that is the reason for the decrease in the efficiency of the cross-bridges. However, the R90P mutation supposedly does not change the efficiency of the cross-bridge work, because the amplitude of the change in the values for Φ_E_ upon the transition of the myosin heads from the weak to the strong binding with F-actin during the ATPase cycle (between the weak binding in the presence of MgATP and the strong binding in the presence of MgADP at a high Ca^2+^) is similar for the R90P-Tpm-BDM and the R90P-Tpm (Figure 10a).

Thus, BDM can induce a marked restoration of the Ca^2+^-dependent regulation of actin–myosin interaction by the troponin–tropomyosin complex, i.e., restores the ability of troponin to switch actin monomers off and to allow the mutant Tpm shifting towards the blocked position. Therefore, the muscle fibers restore their ability to relax at a low Ca^2+^ under the influence of MgATP. The latter is very important, as the appearance of the cross-bridges strongly bound with F-actin at relaxation (so-called rigor-like myosin heads) can cause contracture and can contribute to the development of destructive changes in the muscle tissue [19].

**Figure 11:**
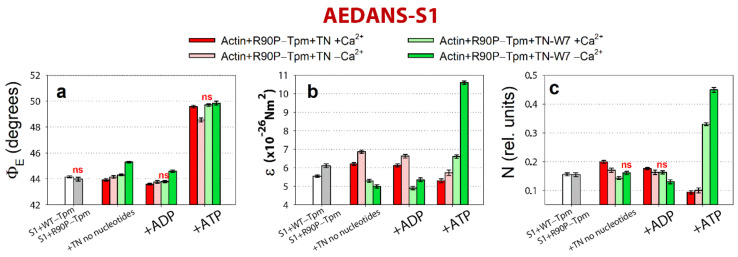
The effect of troponin and Ca^2+^ on the values of Φ_E_ (**a**), ε (**b**), and N (**c**) of the polarized fluorescence of 1,5-IAEDANS bound to S1 (AEDANS-S1), in the absence and in the presence of 100 µM W7, revealed in ghost fibers, containing R90P-Tpm under conditions simulating the sequential steps of the actomyosin ATPase cycle. The experimental conditions and designations are described in Materials and Methods. The data represent the mean values for 5–7 fibers for each experimental condition. The Φ_E_, ε, and N values in the absence of troponin and in the presence of Ca^2+^ and nucleotides are significantly altered by W7 (*p* < 0.05). Index “ns” indicates statistically insignificant differences in the values for Φ_E_ and N between the WT and mutant tropomyosin. Error bars indicate ± SEM.

A similar effect on Ca^2+^ regulation of the contraction in the fibers containing R90P-Tpm can be induced by 0.1 mM W7 (Figure 11).

**Figure 12:**
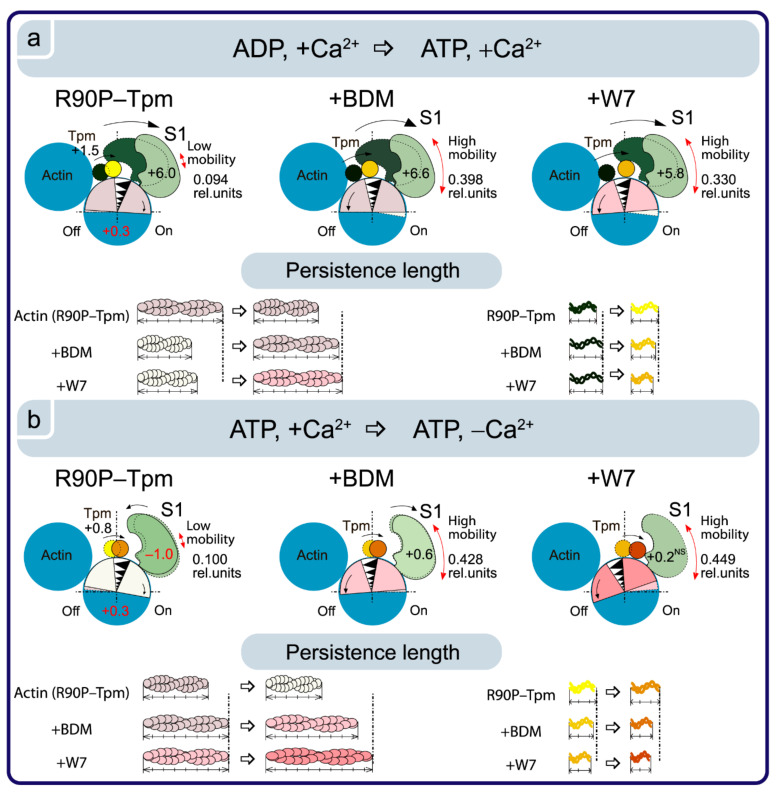
The effect of BDM and W7 on the changes in the R90P-Tpm position, in actin–myosin conformational rearrangements, and in the persistence lengths of the Tpm strands and actin filaments. The value for ε is the bending stiffness. The upper and bottom panels show the transition of the actin-Tpm-TN-S1 complex from the state in the presence of ADP at a high Ca^2+^ to the state in the presence of ATP at a high Ca^2+^ (**a**) and further to the state at a low Ca^2+^ concentration (**b**). BDM stimulates the formation of strong myosin binding with actin in the presence of ADP and the formation of weak myosin binding with actin under the relaxing conditions. W7 does not affect the conformation of the myosin head at a high Ca^2+^, however, it allows for the weak binding of the myosin heads to actin in the presence of ATP at a low Ca^2+^. The mobility of the myosin heads is increased significantly in the presence of BDM and W7, as follows, from the changes in the number of disordered probes (parameter N). The changes in the actin rotation and in the Tpm position are derived from the data on the myosin conformational changes. BDM and W7 increases the persistence length of actin (drawn to scale), and decreases that of Tpm. The designations are the same as in Figure 5. The changes in Φ_E_ for S1 upon transition between the states are shown by numbers.

At a high Ca^2+^, the amount of myosin heads strongly bound to F-actin decreased (the Φ_E_ values did not change or increased), and the bending stiffness (ε) and flexibility of the myosin head attachment to F-actin (N) decreased, except for an increase in the presence of MgATP (Figure 11). In addition, in the presence of W7, the amplitude of change in the Φ_E_ value at transition of the myosin heads from the weak to the strong binding with F-actin during the ATPase cycle (between the weak binding in the presence of MgATP and the strong binding in the presence of MgADP at a high Ca^2+^) was 5.8° for the R90P-Tpm-BDM, which is smaller than the amplitude observed for the R90P-Tpm (6.0°, Figure 12a).

This implies that W7 slightly decreases the effectivity of the cross-bridge work. However, at a low Ca^2+^, W7 extremely decreases the amount of the myosin heads strongly bound to F-actin (the values for Φ_E_ increased, Figure 12a), and produces a noticeable increase in the persistence length of actin filaments and in the flexibility of the myosin head attachment to actin when modeling the muscle fiber relaxation (the values for N increased by 78%, Figure 11c). Thus, W7 can at least partially restore the balance between the strongly- and weakly-bound myosin heads during the ATPase cycle, which is necessary for normal contractility and relaxation in muscle fibers.

## 3. Materials and Methods

### 3.1. Using of Experimental Animals

All of the experiments were performed on skinned muscle fibers and proteins from the skeletal muscles of rabbit (*Oryctolagus cuniculus*). The animals were killed in accordance with the official regulations of the community council on the use of laboratory animals by the methods described earlier [8,9,10,11]. The study was approved by the Animal Ethics Committee of the Institute of Cytology of the Russian Academy of Science (Assurance Identification number F18-00380, valid until 31 October 2022).

### 3.2. Preparation of Proteins and Their Labeling by Fluorescent Probes

Skeletal myosin and actin from fast rabbit muscles were prepared according to Margossian and Lowey [55] and Spudich and Watt [56], respectively. Myosin subfragment-1 (S1) was prepared by α-chymotrypsin digestion of rabbit myosin [57]. The reactive residue Cys707 of S1 was modified with 1,5-IAEDANS [58]. The recombinant γγ-WT-Tpm (control protein containing no mutations) and the R90P-mutant Tpm were obtained by using molecular genetics methods in the bacterial expression system of *E. coli*, as described earlier [59,60]. All of the tropomyosins had an N-terminal extension of two additional amino acids (AlaSer), which compensated for the reduced affinity of recombinant non-acetylated skeletal tropomyosin to F-actin [59]. The labeling of tropomyosin with 5-IAF at Cys190 was performed as described previously [8,27]. The purity of the proteins was examined by SDS-PAGE.

### 3.3. Determination of Actin-Activated ATPase of Myosin Subfragment-1

The rate of the ATPase reaction was determined for the fully regulated reconstituted thin filaments in a solution containing 4 μM F-actin, 0.5 μM S1, 1.25 μM troponin complex, and 1.5 μM WT-Tpm or R90P-Tpm in the following buffer (pH 7.0): 15 mM MOPS, 3 mM MgCl_2_, 1 mM DTT, 1 mM Ca^2+^/EGTA buffer system, with varying Ca^2+^ concentrations ranging from pCa 9.0 to 4.5 at 25 °C. The amount of inorganic phosphate formed was determined by the method of Fiske and Subbarrow [61]. Three experiments were conducted for each experimental condition. Statistical processing of data, calculation of the pCa_50_ value, and plotting were carried out using GraphPad Prism 5.0 software. There are average values of pCa_50_ from three to four independent experiments. Error bars indicate ± SEM.

### 3.4. Preparation and Labeling of Ghost Fibers

The models of the striated muscle fibers were obtained from *m. psoas* of rabbit. Bundles of about 100 fibers were placed into a cooled solution containing 100 mM KCl, 1 mM MgCl_2_, 67 mM K,Na-phosphate buffer, pH 7.0, and 50% glycerol. Single fibers were gently isolated from the glycerinated muscle bundle and incubated during 70–90 min in the solution containing 800 mM KCl, 1 mM MgCl_2_, 10 mM ATP, and 6.7 mM K,Na-phosphate buffer, at pH 7.0 [8]. Thin filaments were reconstructed with Tpm (WT-Tpm or R90P-Tpm) and TN and decorated with S1 by incubating of the fiber in a solution containing 50 mM KCl, 3 mM MgCl_2_, 1 mM DTT, and 6.7 mM K,Na-phosphate buffer, at pH 7.0, and the corresponding proteins. The proteins that did not bind with F-actin were removed by washing the fiber in the same solution without proteins. FITC-phalloidin was dissolved in methanol and conjugated with the F-actin of the fibers, as described before [8,9,10,11].

The final composition of the fibers was examined using 12% SDS-PAGE gels, stained with Coomassie brilliant blue R (Sigma-Aldrich) and scanned in Bio-Rad ChemiDocTM MP Imaging system (Hercules, Contra Costa, CA, USA) (Figure 1). Then, 8–10 fibers were applied to each lane. The excess of proteins were removed by 60 min flushing of the fibers in the washing solution containing 67 mM K, Na-phosphate buffer, 100 mM KCl, and 1 mM MgCl_2_. The ratio of WT-Tpm to the mutant Tpm that bound to the actin was determined in 12% gel by ImageJ 1.48 software.

### 3.5. Polarized Fluorescence Measurements

Steady-state polarized fluorescence was measured in ghost fibers using a flow-through chamber and a polarized fluorimeter, as described before [32]. Fluorescence from the 1,5-IAEDANS-labeled S1 (AEDANS-S1) was excited at 407 ± 5 nm, and from 5-IAF-labeled Tpm (AF-Tpm) and FITC-labeled actin (FITC-actin) at 489 ± 5 nm; the intensity of the fluorescence (I) was recorded in the range of 500–600 nm. The probes in ghost fibers were excited by a 250 W mercury lamp DRSH-250 [10]. The exciting light was passed through a quartz lens and a double monochromator, and was split into two polarized beams by a polarizing prism. The ordinary polarized beam was reflected at the dichroic mirror and was condensed by a quartz objective (UV 58/0.80) on a fiber in the cell during the microscope stage. The emitted light from the fiber was collected by the objective and was led to a concave mirror with a small hole. After passing through the lens and a barrier filter, the beam was separated by a Wollaston prism into polarized beams perpendicular and parallel to the fiber axis. The intensities of four components of polarized fluorescence _‖_I_‖_, _‖_I_⊥_, _⊥_I_⊥_, and _⊥_I_‖_ were detected by two photomultiplier tubes [10,32]. The fluorescence polarization ratios were defined as P_‖_ = (_‖_I_‖_ − _‖_I_⊥_)/(_‖_I_‖_ + _‖_I_⊥_) and P_⊥_ = (_⊥_I_⊥_ − _⊥_I_‖_)/(_⊥_I_⊥_+_⊥_I_‖_). The subscripts ‖ and ⊥ designate the direction of polarization parallel and perpendicular to the fiber axis, the former denoting the direction of the polarization of the incident light and the latter that of the emitted light. In all of the experiments, the background fluorescence intensity of the ghost fiber was 2–3 orders of magnitude less than the fluorescence intensity of the probe specifically associated with the protein, and was taken into account when processing the data.

The experimental data were assessed by a helix-plus isotropic model [32,62,63]. The model is based on the assumption that there are two populations of fluorophores in the muscle fiber: the ordered fluorophores in the amount of (1-N), with their absorption and emission oscillators oriented at the angles Φ_A_ and Φ_E_, respectively, relative to the thin filament axis, and the disordered fluorophores in the amount of N (oriented at the magic angle 54.7°). The number of disordered probes N relates to the mobility of the labeled protein. The motions of the probes relative to the protein are included in the model as the angle γ (the angle between the absorption and emission dipoles). The value of γ is constant for the probes and is assumed to be 17° for 5-IAF bound to tropomyosin, 14° for FITC bound to F-actin, and 20° for 1,5-IAEDANS bound to S1 [10,32]. In this model, the thin filament is assumed to be flexible, i.e., the angle θ between the fiber axis and thin filament is not zero. According to the theory of a semiflexible filament, for a filament length L with one end fixed and the other end free, sin^2^θ = 0.87(kT/ε)L. Thus, the bending stiffness (ε) of the actin filaments can be estimated from sin^2^θ [62].

Measurements were carried out in the washing buffer in the absence of nucleotides (simulating the AM state of the actomyosin complex) or in the presence of 3 mM ADP or 5 mM ATP, mimicking the AM^·ADP and AM**·ATP states, respectively, of actomyosin in the ATPase cycle [8,64]. In the experiments with troponin, the solutions additionally contained either 0.1 mM CaCl_2_ or 2 mM EGTA.

Changes in the polarized fluorescence parameters (Φ_E_, ε and N) were considered as reporting on conformational changes in the protein modified with the probe [8,9,10,11]. The data were obtained from 5−11 fibers (20–55 measurements) for each experimental condition. The statistical significance of the changes in two samples for each experiment (mutant and wild-type tropomyosin, or in the absence and in the presence of BDM/W7) was evaluated using Student’s *t*-test, *p* < 0.05.

## 4. Conclusions

The application of reconstituted muscle fibers has enabled us to reveal some unknown details about the regulation of the actin–myosin interaction by the tropomyosin–troponin complex during the ATPase cycle in the muscle fibers containing the wild-type and R90P-mutant Tpms. Our data have shown that the Ca^2+^ regulation of the actin–myosin interaction is mediated by conformational changes in tropomyosin–troponin complex, actin, and myosin heads, which result in spatial rearrangement and alterations in the persistence length of Tpm and F-actin that presumably cause azimuthal shifting of the tropomyosin strands. The conformational changes in the troponin–tropomyosin complex, F-actin, and the myosin heads initiated by Ca^2+^ and the nucleotides are interdependent [8,9,10,11], therefore a point mutation in any of these proteins should disrupt this interdependency and may induce deregulations of the actin–myosin interaction. Our work demonstrates that the R90P substitution in tropomyosin induces such an uncoupling. Indeed, troponin loses its ability to move Tpm strands towards the outer domains of actin and to switch actin monomers off at a low Ca^2+^ (Figure 4 and Figure 7). This may contribute to the high Ca^2+^-sensitivity that we observed in protein solution (Figure 2). Furthermore, the R90P mutation also may alter the ability of Tpm to control the formation of the strong binding of the myosin heads to F-actin throughout the ATPase cycle; the amount of myosin heads strongly bound to F-actin when mimicking the AM and AM∙ADP stages decreases (Figure 8), therefore the actin-activated ATPase activity of S1 decreases (Figure 2) and muscle weakness is observed [19].

It is known that the substitution of positively charged Arg90 with Pro residue that disrupts the coiled-coil structure dramatically destabilizes not only the N-terminal part of the Tpm molecule where it is located, but also its C-terminal part [28], and, as a result, partially destabilizes the tropomyosin molecule in the region of the site for Tpm binding to troponin T. The alteration in the interaction of Tpm with troponin T can result in the disruption of the ability of troponin to switch the thin filaments on and off. This can lead to the inhibition of the ATPase activity at a high Ca^2+^ (i.e., to a decrease in force production) and an increase in the Ca^2+^-sensitivity and the appearance of the rigor-like myosin heads, which strongly bind to F-actin at relaxation (Figure 8). The rigor-like myosin heads were observed in our earlier studies of other mutant Tpms, which are associated with arthrogryposis, congenital muscle fiber type disproportion, and cap myopathy. Similar rigor-like myosin heads were found by us when mimicking relaxation in the muscle fibers containing the point mutations: E139X in β-Tpm [9], R91G in β-Tpm [44], R168G in α-Tpm [26], A155T [45], and E173A [11] in γ-Tpm. Therefore, it seems important to reduce the effect of R90P mutation, for which we used the inhibitor of the ATPase activity of myosin, BDM, and the Ca^2+^-desensitizer, W7. It has been shown that both inhibitors allow for the mutant Tpm moving towards the blocked position, restore the ability of troponin to switch actin monomers off at a low Ca^2+^, and reduce the amount of the rigor-like myosin heads upon relaxation. The results show the promise for the use of the studied chemical compounds to partly rehabilitate the effective myosin cross-bridges work.

Unfortunately, both inhibitors reduce the ATPase activity at a high Ca^2+^ (Figure 9). Furthermore, one of the main properties of compounds that are promising for the treatment of skeletal muscle pathologies is the specificity of the interaction with skeletal muscle myosin or troponin, and the absence of an effect on the cardiac contractility. BDM inhibits skeletal muscle myosin II and it does not inhibit the ATPase activity of the other myosins, but affects many other proteins and processes independent of myosin ATPase activity [46,49]. W7 reversibly inhibits ATPase and tension in both skeletal and cardiac fibers, resulting in a reduced calcium sensitivity and cooperativity of ATPase and tension activations [54]. Further research is needed to develop compounds with a suitably high specificity for the skeletal muscle isoforms, and to test their action in model muscle fibers, in model animals, and, with successful effects, in preclinical and clinical trials. A successful example of a cardiac myosin inhibitor that decreases the abnormal number of actin-binding myosin heads transitioning from the weakly to the strongly bound state is Mavacamten. It is under investigation in clinical trials (phases 2 and 3) in adults with hypertrophic cardiomyopathy (obstructive and non-obstructive). Mavacamten showed its selectivity for cardiac myosin with which its effect on Ca^2+^-sensitivity was four-fold higher than with skeletal myosin [65].

Investigation of the molecular mechanisms of congenital myopathy at the earliest stages of the development of the disease allows for determining the targets for therapeutic action, and selecting the pharmacological agents capable of restoring normal function. As congenital myopathy manifests, as a rule, from birth or in early childhood, the timely initiated etiotropic treatment of the disease is critically important for preventing or slowing the progression of muscle weakness and hypotension, manifestation of compensatory processes, including the appearance of intracellular inclusions, as well as further consequences of impaired contractile and motor function, such as respiratory and heart failure.

## Data Availability

The data presented in this study are available on request from the corresponding author.

## Abbreviations

Tpm	Tropomyosin
WT-Tpm	Wild-type tropomyosin
R90P-Tpm	Tropomyosin with the Arg90Pro substitution
TN	Troponin
S1	Myosin subfragment-1
1,5-IAEDANS	N-(iodoacetaminoethyl)-1-naphthyl-amine-5-sulfonic acid
5-IAF	5-Iodoacetamidofluorescein
FITC	Fluorescein isothiocyanate
W7	(N-(6-minohexyl) 5-chloro-1-napthalenesulfonamide)
BDM	2, 3-Butanedione 2-monoxime
